# Mechanism Underlying Acupuncture Therapy in Spinal Cord Injury: A Narrative Overview of Preclinical Studies

**DOI:** 10.3389/fphar.2022.875103

**Published:** 2022-04-07

**Authors:** Kunpeng Jiang, Yulin Sun, Xinle Chen

**Affiliations:** ^1^ Department of Hand and Foot Surgery, Zhejiang Rongjun Hospital, Jiaxing, China; ^2^ Department of Neurosurgery, Zhejiang Rongjun Hospital, Jiaxing, China

**Keywords:** acupuncture, spinal cord injury, therapy, mechanism, apoptosis, inflammation, oxidative stress, neuroprotection

## Abstract

Spinal cord injury (SCI) results from various pathogenic factors that destroy the normal structure and function of the spinal cord, subsequently causing sensory, motor, and autonomic nerve dysfunction. SCI is one of the most common causes of disability and death globally. It leads to severe physical and mental injury to patients and causes a substantial economic burden on families and the society. The pathological changes and underlying mechanisms within SCI involve oxidative stress, apoptosis, inflammation, etc. As a traditional therapy, acupuncture has a positive effect promoting the recovery of SCI. Acupuncture-induced neuroprotection includes several mechanisms such as reducing oxidative stress, inhibiting the inflammatory response and neuronal apoptosis, alleviating glial scar formation, promoting neural stem cell differentiation, and improving microcirculation within the injured area. Therefore, the recent studies exploring the mechanism of acupuncture therapy in SCI will help provide a theoretical basis for applying acupuncture and seeking a better treatment target and acupuncture approach for SCI patients.

## Introduction

Spinal cord injury (SCI) causes structural and functional damage through direct or indirect factors, leading to motor, sensory, and autonomic nerve dysfunction (McDonald and Sadowsky, 2002). The global incidence of SCI ranges from 3.6 to 195 per 1,000,000 (Jazayeri et al., 2015). In China, the incidence of traumatic SCI was standardized to 49.8 per 1,000,000 per year based on the 2010 census, and the mean age of patients at the time of injury was 43.7 ± 17.1 years (Jiang et al., 2021). SCI is a common cause of death and disability, with severe neurological dysfunction and complications, including neuropathic pain, pressure ulcers, and urinary tract infection. In addition, it causes a substantial psychological and social burden on patients, families, and the society (Wannapakhe et al., 2015; Gedde et al., 2019; Moshi et al., 2021). Pathophysiological changes after SCI include primary and secondary injuries. Compared with the unpredictability of primary injury, the underlying mechanism and effective treatment of secondary injury is the primary focus of the current SCI research (Belegu et al., 2007; Jeong et al., 2021). SCI is a dynamic pathological process causing nerve cell and nerve fiber edema at the initial stages, followed by microcirculation disorders due to damaged blood cells (Rivlin and Tator, 1978; Tator and Fehlings, 1991). Then, the nerve cell axons degenerate or die and are gradually replaced by glial cells (O’Shea et al., 2017; Lukacova et al., 2021). Aggravation of cellular, molecular, and other factors at different stages post SCI leads to a series of pathophysiological changes, reducing the spinal cord functional recovery (Tator, 1995). Timely and efficient intervention can partially stimulate potential nerve cells and axonal regeneration and resume the function of axons and neurons. The current treatments for SCI mainly include surgery, medication, and behavioral, physical, and supportive therapies (Becker et al., 2003; Boulenguez and Vinay, 2009; Ramer et al., 2014).

Acupuncture is a substantial alternative and adjunctive therapy for SCI and is a vital component of traditional Chinese medicine. Electroacupuncture, a method based on acupuncture combined with the micro-current wave of bioelectricity, was developed by combining traditional and modern medicines. In recent years, acupuncture–electroacupuncture has been widely used in clinical practices and exerts a significant neuroprotective effect against SCI and its complications (Paola and Arnold, 2003; Shin et al., 2009; Ma et al., 2015; Fan et al., 2018; Lu et al., 2020). Compared with other therapeutic methods, acupuncture is non-toxic and has a simple operation and low cost, but its mechanism remains unclear. This article summarizes the potential mechanism of acupuncture in SCI to provide the updated theoretical basis depicting various clinical applications of acupuncture in SCI patients (Table 1).

**TABLE 1:** Summary of preclinical studies of acupuncture therapy in spinal cord injury in recent 5 years.

Ref	Species	Acupuncture therapy	Outcome	Mechanism
Wang X et al. (2021)	Male SD rats	EA at *Dazhui* (GV14) and *Mingmen* (GV4) for 20 min daily until they were euthanized	Improve neurological function and promote the repair of the injured spinal cord tissue	Inhibit the Notch signaling pathway and regulate the downstream protein expressions (Delta1, Presenilin1, Hes1, and Hes5)
Dai et al. (2021)	Female C57BL/6 mice	EA at *Zusanli* (ST 36) and *Sanyinjiao* (SP 6) for 10 min daily for 6 days, followed by 1 day off and last for 4 weeks	Improve hindlimb motor function and protect neurons and myelinated axons	Inhibit inflammatory response and oxidative stress through activating the ApoE and Nrf2/HO-1 signaling pathway
Hu et al. (2021)	Female SD rats	EA at *Jiaji* (EX-B2) for 30 min daily for 2 weeks	Promote the recovery of spinal cord nerve function	Inhibit the expression of pro-inflammatory cytokines such as IL-1β, IL-6, and TNF-α and the Nogo-NgR signaling pathway
Yang et al. (2021)	Female SD rats	EA at *Zhiyang* (GV9), *Jizhong* (GV6), *Yaoshu* (GV2), and *Changqiang* (GV1) twice a day for 8 weeks	Accelerate neural network reconstruction and restoration of spinal cord function	Increase the local production of NT-3, improve the hostile microenvironment of the injured spinal cord by dampening local inflammation, and foster the biological functions of MSC-derived neuron-like cells
Hongna et al. (2020)	Female SD rats	EA at *Jiaji* (EX-B2) for 30 min daily until they were euthanized	Improve locomotor function	Regulate autophagy flux and inhibit necroptosis
Lu et al. (2020)	Male SD rats	EA at *Ciliao* (BL32) and *Zhongliao* (BL33) for 20 min daily for 10 days	Improve neurogenic bladder (the *Ciliao* acupoint is superior to the *Guanyuan* point)	Reduce histomorphological abnormalities in interstitial cells of Cajal and inhibit the expression of hyperpolarization-activated cyclic nucleotide-gated channel proteins
Hu et al. (2020)	Male SD rats	EA at *Jiaji* (EX-B2) for 20 min daily for 7 or 14 days	Promote the recovery of the motor function	Affect the plasticity of peripheral nerve networks by regulating the Semaphorin 3A signal
Xu H et al. (2021)	Female SD rats	EA at *Zhiyang* (GV9), *Jizhong* (GV6), *Yaoshu* (GV2), and *Changqiang* (GV1) twice a day for 2 weeks	Promote the survival, axonal regrowth, and synaptic maintenance of spinal cord neurons	Trigger the synthesis and secretion of NT-3 by activating the CGRP/RAMP1/calcium/αCaMKII pathway
Cheng et al. (2020)	Male SD rats	EA at *Dazhui* (GV14) and *Mingmen* (GV4) for 30 min daily for a week	Improve functional recovery	Inhibit the phosphorylation of JNK/p66^Shc^-mediated oxidative stress and reduce the p38MAPK-mediated microglial activation and inflammatory reaction
Zhou et al. (2020)	Male SD rats	EA at *Dazhui* (GV14), *Mingmen* (GV4), and *Jiaji* (EX-B2) for 20 min twice daily for 3 weeks	Improve hindlimb motor function	Twenty-nine upregulated and 139 downregulated miRNAs in the EA group. The MAPK, Wnt, and NF-κB signaling pathways are involved
Ding et al. (2020)	Male SD rats	Acupuncture combined with moxibustion at *Dazhui* (GV14), *Jiaji* (EX-B2), *Yaoyangguan* (GV3), *Zusanli* (ST36), and *Ciliao* (BL32) for 30 min daily for 7 or 14 days	Recover motor function, preserve the neuron cells, and alleviate the apoptosis of nerve cells	Improve the mRNA and protein levels of Shh and Gli-1
Li et al. (2020)	Male SD rats	EA at *Dazhui* (GV14) and *Mingmen* (GV4) for 20 min daily until they were euthanized	Improve locomotor function	Affect cell growth, apoptosis, and autophagy through the PI3K/AKT/mTOR signaling pathway
Song et al. (2022)	Male SD rats	EA at *Zusanli* (ST36) for 20 min daily until they were euthanized	Promote the recovery of neurological function	Stimulate ascending peripheral nerve conduction
Xiao et al. (2019)	Female SD rats	EA at *Yaoyangguan* (GV3), *Dazhui* (GV14), *Zusanli* (ST36), and *Ciliao* (BL32) for 20 min daily for 2 weeks	Promote axonal regeneration	Inhibit the Nogo/NgR and Rho/ROCK signaling pathway
Hong et al. (2021)	Male SD rats	EA at *Yaoyangguan* (GV3), *Dazhui* (GV14), *Zusanli* (ST36), and *Ciliao* (BL32) 20 min daily for 2 weeks	Improve lower limb movement function and spinal cord tissue morphology	Reduce mRNA and protein expression of RhoA and ROCKII, decrease p-MLC protein expression and p-MLC/MLC ratio, and suppress the cPLA2 activity and PGE_2_ level
Xu et al. (2019)	Female SD rats	Fire needle at *Jiaji* (EX-B2) in 1/3 s daily	Improve lower limb locomotor function	Promote endogenous NSC proliferation differentiating into neurons by promoting the activation of Wnt/β-catenin and inhibiting the overexpression of ERK.
Prado et al. (2019)	Dog	EA at GV2, DU20, GV3a, and GV6; bilateral BL19, BL23, and BL24; unilateral KI3, ST36, LV3, and *Wei Jian* for 20 min three times a week for the initial 7 weeks and two times a week for 5 more weeks	Improve neurological function	None
Jin et al. (2019)	Female SD rats	EA at *Zhiyang* (GV9), *Jizhong* (GV6), *Yaoshu* (GV2), and *Changqiang* (GV1) daily for 8 weeks	Improve locomotor function	Enhance the survival and synaptic integration of grafted NT-3 and TRKC gene-overexpressing neural stem cell-derived neural network scaffold with the host spinal neural network by increasing the NT-3 level and activating the NT-3/TRKC/AKT signaling pathway
Alvarado-Sanchez et al. (2019)	Female Long–Evans rats	EA at *Mingmen* (GV4) per 30 min until they were euthanized	Improve motor function recovery and the amount of preserved spinal cord tissue	Decrease oxidative stress and lipid peroxidation
Zhang et al. (2019)	Female SD rats	Sacral EA intervention for 7 days	Inhibit apoptosis, protect nerve cells, promote the coordination of micturition reflex, and improve neurogenic bladder function	Improve the expressions of both NGF/TrkA signaling and Akt signaling
Wei et al. (2018)	Female C57BL/6 mice	EA at *Jiaji* (EX-B2) for 15 min for 5 days, followed by 1 day off and last for 4 weeks	Restore locomotor function	Inhibit the expression of PTEN and p53 and increase the levels of pmTOR/Akt/Erk and myelin basic protein
On-Ong-Arj et al. (2018)	Male Wistar rats	Yellow laser acupuncture at *Yaoshu* (GV2) for 10 min at 15 min, 6, 12, and 24 h after SCI on the first day, followed by 10 min daily for 7 days	Improve both motor deficit and neurodegeneration in the ventral horn of the spinal cord	Increase the expression of BDNF and inhibit inflammation, apoptosis, and oxidative stress
Wang et al. (2019)	Male Wistar rats	EA at *Neiguan* (PC6) and *Jianshi* (PC5)	Alleviate SCI-induced neuropathic pain	Inhibit the PI3K-mTOR signaling pathway
Wang et al. (2018)	Female Wistar rats	EA at *Dazhui* (GV 14) and *Baihui* (GV20) for 15 min daily for 2 weeks	Improve the recovery of nerve movement	Reduce the expression of platelet-activating factor and caspase-9 protein
Li et al. (2018)	Female Wistar rats	EA at *Jiaji* (EX-B2), *Mingmen* (GV4), and *Dazhui* (GV14) for 15 min daily for 6 days. After a 2-day interval, the second course started, with three courses in total.	Enhance the growth of nerve fibers and improve the hindlimb motor function recovery	None
Tu et al. (2018)	Male SD rats	EA at *Zusanli* (ST-36) and *Yanglingquan* (GB-34) performed between 09:00 and 11:00 daily for 7 days	Reduce mechanical allodynia and thermal hyperalgesia	Inhibit the activation of spinal microglia and block the BDNF-TrkB signaling pathway
Wang et al. (2017)	SD rats	EA at *Zusanli* (ST-36)-*Xuanzhong* (GB39) and *Futu* (ST32)-*Sanyinjiao* (SP6) for 30 min until they were euthanized	Improve hindlimb locomotor and sensory function	Systematic regulation of neurotrophic factors and their receptors
Tu et al. (2017b)	Male SD rats	EA at *Baihui* (GV20) and *Fengfu* (GV16) or *Dazhui* (GV14) and *Mingmen* (GV4)	EA stimulation at GV14 and GV4 promote the recovery of locomotor function	Improve mRNA and protein expression of BDNF and NT-3
Nascimento de Souza et al. (2017)	Male Wistar rats	Bee venom at a dose of 0.08 mg/kg injected subcutaneously at *Zusanli* (ST36) and *Yaoyangquan* (GV3) (20 μL at each point) once immediately after SCI and 24 h, 7, and 14 days after SCI.	Induce locomotor recovery	Reduce the expression of IL-6 and increase the expression of IL-10
Zhang J et al. (2017)	Male SD rats	EA at *Dazhui* (GV14) and *Mingmen* (GV4) for 20 min daily for 2 weeks	Promote spinal recovery	Promote the differentiation of neural stem cells into spinal neurons by enhancing Wnt1/β-catenin signaling
Zhu et al. (2017)	Male SD rats	EA at *Jizhong* (GV6) and *Zhiyang* (GV9) 30 min daily for 7 days	Promote the proliferation of neural stem cells and the survival of neurons	Promote the expression of neuronal markers Nestin, NeuN, and CGRP and inhibit cellular apoptosis and inflammation by downregulating miR-449a
Tu et al. (2017a)	Male SD rats	EA at *Dazhui* (GV14) and *Mingmen* (GV4) for 30 min at 30 min, 12, and 24 h after SCI.	Improve hindlimb locomotor function	Decrease the mRNA and protein expression of the subunits of NMDAR NR1 and NR2A
Liu and Wu (2017)	Female SD rats	EA at *Jizhong* (GV6) and *Zhiyang* (GV9) for 20 min daily for a week	Improve functional recovery and inhibit neuronal apoptosis	Reduce Bax and inhibit the sodium channel Nav1.3 expression by regulating miR-214
Zhao et al. (2017)	Male SD rats	EA at *Jizhong* (GV6) and *Zhiyang* (GV9) for 20 min every other day for 4 weeks	Improve motor function	Enhance the expression of IL-10, M2 marker CD206, NT-3, and the proportion of M2 macrophages
Escobar-Corona et al. (2017)	Male Wistar rats	EA at *Huantiao* (GB30), *Yinmen* (BL37), *Jizhong* (GV6), and *Zhiyang* (GV9) for 40 min every other day for 4 weeks	Improve gait locomotion, H-reflex, and ventral root potential	None

Abbreviations: αCaMKII: calmodulin-dependent protein kinase; BDNF: brain-derived neurotrophic factor; CGRP: calcitonin gene-related peptide; EA: electroacupuncture; MSC: mesenchymal stem cell; NMDARs: N-methyl-D-aspartate (NMDA) receptors; NSCs: neural stem cells; NT-3: neurotrophin-3; RAMP: receptor activity-modifying protein; SCI: spinal cord injury.

## Mechanism of Acupuncture Therapy in Spinal Cord Injury

### Reduction of Oxidative Stress

Free radicals can be generated and released after SCI. While the degree of oxidation exceeds the ability of the antioxidant system, excessive free radicals will initiate the oxidation chain reaction (Bringans et al., 2022). Reactive oxygen species (ROS) and reactive nitrogen species (RNS) can efficiently react with intracellular macromolecules, causing cell death and tissue damage and subsequently aggravating SCI. The spinal cord contains many polyunsaturated fatty acids, thus making it sensitive to oxidative stress. The spinal cord neurons have active oxidative metabolism but low antioxidant capacity, making neurons and glial cells significantly vulnerable to oxidative stress. Hence, reactive oxygen metabolites accumulate, resulting in excessive consumption of antioxidants from tissues after SCI (Genovese and Cuzzocrea, 2008; Figueroa et al., 2013; Lim et al., 2013; Wojdasiewicz et al., 2020).

Superoxide dismutase (SOD) is an active protease scavenging free radicals and protecting cells from oxidative damage. It eliminates the oxidation products produced after SCI. The SOD level reflects the ability to clear free radicals and has a vital role in balancing oxidation and antioxidation. Malondialdehyde (MDA) is a lipid peroxidation metabolite, reflecting the degree of oxidative stress (Woźniak et al., 2016; Wu et al., 2017). The lipid peroxidation can interfere with Ca^2+^ transport from the cell membrane by inhibiting the Ca^2+^-ATPase activity, causing intracellular Ca^2+^ overload and enhanced ion imbalance (Rohn et al., 1993; Rohn et al., 1996). In addition, oxidative stress post SCI destroys ion homeostasis both inside and outside the membrane. Moreover, abundant Ca^2+^ enters and accumulates within the mitochondria, leading to mitochondrial destruction, aerobic energy metabolism dysfunction, and inhibition of ATP synthesis (Brown et al., 2006; Visavadiya et al., 2013; Scholpa and Schnellmann, 2017). Studies have revealed that acupuncture, electroacupuncture, and laser acupuncture can reduce oxidative stress after SCI (Wu et al., 2002; On-Ong-Arj et al., 2018; Alvarado-Sanchez et al., 2019). The results from a traumatic SCI model study showed that electroacupuncture at GV26 reduces radical hydroxyl concentration and increases lipid peroxidation. At the same time, stimulation of GV4 decreases oxidative stress and improves motor function recovery in the hind limbs of rats with paralysis, indicating electroacupuncture at GV4 could be a therapeutic alternative (Juarez Becerril et al., 2015). Jiang et al. found that electroacupuncture at *Shuigou* (DU26) and *Fengfu* (DU16) acupoints induce antioxidation effects by enhancing the SOD activity and decreasing the MDA level (Jiang et al., 2014). Similar to the effect of the reactive oxygen species (ROS) scavenger, acupuncture can inhibit superoxide anion production, decrease JNK/p66Shc-mediated ROS generation, upregulate the apolipoprotein E (ApoE) and nuclear factor E2-related factor 2 (Nrf2)/heme-oxygenase-1 (HO-1) signaling pathways, and reduce the ROS-induced p38MAPK and ERK activation in microglia after SCI (Choi et al., 2012; Dai et al., 2021). Notably, the inhibitory effect of electroacupuncture on p38MAPK is significantly enslaved to the acupuncture frequency (Cheng et al., 2020).

### Inhibition of Neuronal Apoptosis

Apoptosis, predominantly neuronal apoptosis, is an essential pathological mechanism causing secondary spinal cord injury (Abbaszadeh et al., 2020; Shi et al., 2021). Axonal injury and neuronal apoptosis block nerve conduction pathways after SCI and aggravate secondary injuries. Therefore, inhibition of apoptosis can induce SCI recovery (Beattie, 2004). The anti-apoptotic mechanisms of acupuncture have been widely explored. Acupuncture protects the nerves and reduces apoptosis of neurons and oligodendrocytes, thus improving functional recovery after SCI (Cai and Shen, 2018). In addition, electroacupuncture can inhibit spinal cord neuronal apoptosis by increasing the Bcl-2 expression and inhibiting caspase-3 and Bax (Zhao et al., 2008; Shi et al., 2016; Liu and Wu, 2017; Zhu et al., 2017).

Poly-ADP ribose polymerase (PARP) is the most significant substrate of caspase-3, and activated PARP can cause apoptosis mediated by apoptosis-inducing factor (AIF) (Kang et al., 2004). Previous studies showed that electroacupuncture could ameliorate early brain injury after subarachnoid hemorrhage by inhibiting the PARP-1/AIF pathway (Lang et al., 2020). Moreover, electroacupuncture also reduces the PARP expression in cerebral ischemia/reperfusion and Parkinson’s disease (Sun et al., 2003; Yu et al., 2020). Furthermore, Liu et al. found that apoptosis post SCI was accompanied by cleaved PARP upregulation and electroacupuncture treatment attenuation (Liu and Wu, 2017).

BNIP3 is a member of the Bcl-2 family that induces apoptosis by promoting mitochondrial permeability transport pore opening and mitochondrial damage (Yu et al., 2018). In addition, the BNIP3 expression is elevated in rats after SCI (Yu et al., 2018), and electroacupuncture at GV20-GB7 reduced BNIP3 after intracerebral hemorrhage (Guan et al., 2021).

Heat shock protein (HSP) is an endogenous stress protein with various biological protective effects. HSP family members such as HSP 70 and HSP 72 have a protective effect on neurons after SCI (Chang et al., 2014; Xu et al., 2021a; Kim et al., 2022). Acupuncture has been demonstrated to have a neuroprotective role in cerebral ischemia by regulating HSP 70 (Xu et al., 2014; Shi et al., 2017). Gao et al. reported that HSP 90 participates in electroacupuncture-induced analgesia in chronic neuropathic pain (Gao et al., 2021). Other signaling pathways, such as PI3K/Akt/Erk, Nogo/NgR, Rho/ROCK, and mTOR, may also include the acupuncture-related beneficial effects against SCI (Renfu et al., 2014; Wei et al., 2018; Xiao et al., 2019; Li et al., 2020).

The toxic effects of excitatory amino acids play an essential role in the pathogenesis of SCI. The glutamate ion receptor activated by the N-methyl-D-aspartate (NMDA) receptor induces excessive Ca^2+^ influx and destroys mitochondrial function, thus stimulating the death of neurons (Xie et al., 2014; Inquimbert et al., 2018). Studies found that electroacupuncture can protect the spinal cord after SCI by reducing the expression of the NMDA receptor subunit NR1 and NR2A in the injured area (Tu et al., 2017a). It can also alleviate mechanical allodynia by inhibiting the upregulation of NR2B after chronic constrictive injury (Zhao et al., 2019).

Recent studies have observed that electroacupuncture can improve the locomotor function by regulating autophagy flux and inhibiting necroptosis after SCI (Hongna et al., 2020). Furthermore, Fang et al. depicted that pre- and post-conditioning electroacupuncture alleviates spinal cord ischemia–reperfusion injury, partly through autophagy upregulation accompanied by apoptosis inhibition (Fang et al., 2017). Moreover, studies conducted in intracerebral hemorrhage rat models show the effect of ferroptosis inhibition by acupuncture (Kong et al., 2021; Li et al., 2022). Therefore, apoptosis, autophagy, necroptosis, and ferroptosis should be clarified in future acupuncture studies on SCI.

### Restrain of Inflammatory Response

After SCI, infiltrating leukocytes attracted by the innate immune response leads to an inflammatory cascade in the area of injury, and an excessive inflammatory response damages the spinal cord tissue. In addition, leukocytes, microglia, astrocytes, and macrophages release many pro-inflammatory cytokines and chemokines, including interleukin-1 (IL-1), IL-6, and tumor necrosis factor-α (TNF-α), which aggravate local inflammation and damage axons and neurons (Zhou et al., 2014a; Tang et al., 2020a; Brockie et al., 2021; Hellenbrand et al., 2021). Therefore, regulating inflammatory factors and improving neuroinflammation is of great significance for the recovery of SCI.

Neuroprotection by acupuncture is partially mediated by inhibiting inflammation and microglial activation after SCI (Choi et al., 2010; Jiang et al., 2014). However, the inflammatory response in SCI has two sides; it exerts a positive reaction against injury and aggravates secondary injury post SCI. The pro-inflammatory macrophage/microglia (M1 subsets) and anti-inflammatory macrophage/microglia (M2 subsets) are significant. Therefore, regulating the polarization of M1 and M2 macrophages/microglia can affect the inflammatory response process after SCI (Buzoianu-Anguiano et al., 2021; Ding et al., 2021; Hashemizadeh et al., 2022). Previous studies have shown that acupuncture can ameliorate SCI by regulating M1 and M2 macrophages (Zhao et al., 2017). It also reduces the release of pro-inflammatory cytokines such as IL-6, TNF-α, nitric oxide synthase, and cycloxygenase-2 (Choi et al., 2010).

The purinergic receptors P2X4 and P2X7 are overexpressed on the cell surface of spinal dorsal horn microglia involved in microglial activation, which significantly contributes to the inflammation after SCI (Deng et al., 2018; Du et al., 2019; Kobayakawa et al., 2019; Song et al., 2022). Electroacupuncture can inhibit P2X7 receptor-mediated microglial activation and attenuate neuropathic pain (Wu et al., 2021a). It can also relieve pain hypersensitivity by inhibiting P2X7 receptor-positive microglia after chronic constriction injury (Xu et al., 2016). In addition, acupuncture reduces diabetic peripheral neuralgia by downregulating the P2X4 expression in rat spinal microglia (Tang et al., 2020b).

The inflammasome is an essential component of host defense response, recognizing pathogen-associated molecular patterns and damage-associated molecular patterns. It mediates the release of pro-inflammatory factors after injury. The family of NOD-like receptors (NLRs) is a vital member of the inflammasome, with NLRP3 being the most studied inflammasome in central nervous system disorders. The ability of acupuncture to attenuate the inflammatory response through inflammasome regulation, especially NLRP3, has been explored in many neurological diseases, including autism (Zhao et al., 2022), postoperative cognitive dysfunction (Sun et al., 2021), depression (Li et al., 2021), Alzheimer’s disease (Jiang et al., 2018; Zhang et al., 2021), cerebral ischemia (Jiang et al., 2019), and vascular dementia (Du et al., 2018). Further research is needed to explore the role of the inflammasome, including NLRs, in acupuncture-induced beneficial effects against SCI.

Choi et al. demonstrated that elevated p38MAPK accelerated the microglial secretion of inflammatory mediators after SCI. Electroacupuncture can effectively downregulate the p38MAPK phosphorylation level, inhibit microglial activation, and promote nerve regeneration (Choi et al., 2010). Hu et al. demonstrated that the combination of gangliosides with electroacupuncture at *Jiaojia* (EX-B2) has a more substantial effect in promoting the recovery of nerve function, which could be related to the inhibition of pro-inflammatory cytokines and the Nogo-NgR signaling pathway (Hu et al., 2021).

### Improvement of Microcirculation Dysfunction

SCI can cause rupture, hemorrhage, and capillary embolism, leading to microcirculation dysfunction. Improved microcirculation can reduce cellular apoptosis and promote functional recovery (Tator and Koyanagi, 1997). Reduced blood flow and intramedullary vasospasm are seen after SCI. Vasoconstriction factors such as endothelin 1 (ET-1), prostaglandin E2 (PGE2), and thromboxane A2 (TXA2) cause vasospasm aggravation and blood flow reduction. As a result, the blood–spinal cord barrier gets disrupted, leading to inflammatory cell infiltration and spinal tissue edema (Tempel and Martin, 1992; Mitsuhashi et al., 1994; McKenzie et al., 1995; Wang et al., 2007; Sinescu et al., 2010).

Clinical studies conducted in healthy adults demonstrated that acupuncture influences the tortuousness of capillary loops, the diameter of the afferent loop, and capillary refill time, thereby regulating the microcirculation (Scardina et al., 2009; Yeh et al., 2021). In animal experiments, acupuncture can also improve the blood flow within the brain after hemorrhage or ischemia. It is primarily associated with the regulation of the vascular endothelial growth factor (VEGF), angiopoietin 1 (Ang-1), Ang-2, angiotensin II type I receptor, endothelin receptor, and EphB4/EphrinB2-mediated Src/PI3K signal pathways (Tian et al., 2013; Zhou et al., 2014b; Wu et al., 2021b). In addition, a study using the intervertebral disc extrusion model revealed that electroacupuncture improves microcirculation characterized by high blood flow, micro-vessel density, and reduced vacuolation within the white matter (Jiang et al., 2015). Acupuncture can also regulate microcirculation and attenuate neurological dysfunction by suppressing the cPLA2 activity and PGE2 level (Hong et al., 2021).

### Attenuation of Glial Scar Formation

Glial cells play an essential role in the physiological function inside the spinal cord microenvironment and induce excessive hyperplasia of the glial scar under pathological conditions. On the one hand, a glial scar can limit the lesion expansion and protect the surrounding tissues from injury. On the other hand, it restricts neuronal regeneration (Faulkner et al., 2004; Pekny et al., 2014; Tran et al., 2018; Gu et al., 2019). During the spinal cord recovery, astrocytes proliferate and secrete a variety of extracellular matrices to form a glial scar, hindering the neural pathway recovery. The significant molecules participating in glial scar formation are chondroitin sulfate proteoglycans (CSPGs) and keratan sulfate proteoglycans produced by astrocytes (Zhang et al., 2006; Wang et al., 2021a; Tran et al., 2021). CSPG accumulation at the injured area inhibits the axonal growth, and reducing the CSPG expression can promote axonal regeneration and remyelination (Siebert and Osterhout, 2011). Electroacupuncture can downregulate the CSPG protein expression and stimulate axonal regeneration, leading to structural and functional recovery after SCI (Ding et al., 2011). It also stimulates the differentiation of transplanted bone marrow mesenchymal stem cells (MSCs) and promotes corticospinal tract regeneration across injured sites in the caudal cord, with CSPG protein involvement (Ding et al., 2013). Numerous studies have shown that acupuncture can restrict astrogliosis and alleviate neurological dysfunction caused by diseases such as hydrocephalus (Tida et al., 2018) and cerebral ischemia (Han et al., 2010; Tao et al., 2016; Young-Wook et al., 2019).

Glial fibrillary acidic protein (GFAP) is a crucial component of astrocytes. As an important marker of glial scar formation, GFAP depicts the proliferative state of astrocytes (Brenner, 2014; Yang and Wang, 2015). In addition, GFAP secreted by astrocytes forms a physical barrier to isolate damaged tissue, provides mechanical strength, and limits axonal growth due to the physical barrier (Pekny et al., 2014). Fire needle acupuncture and electroacupuncture can decrease the GFAP expression, leading to the differentiation of neural stem cells (NSCs) and inhibition of astrocyte activation, respectively (Zhang et al., 2018; Xu et al., 2019). Liu et al. observed that electroacupuncture increases the gene and protein expression of GFAP and the platelet-derived growth factor (PDGF) after spinal cord transection, promoting locomotor function recovery (Liu et al., 2013). Interestingly, Wei et al. revealed that electroacupuncture elevates GFAP levels only at the early phase after SCI and reduces the GFAP expression later during recovery (Wei et al., 2017), indicating diverse functionalities of acupuncture in SCI. Choosing the time points and interval of acupuncture therapy exerting a better effect is an important issue that needs to be explored in future studies.

### Promotion of Neural Stem Cell Proliferation and Differentiation

SCI induces damage to the segmentary neurons, axons, and glial cells at the injury site, forming a hole at the center of the spinal cord. The loss of neurons within the injured section and the disruption of the ascending sensory and descending motor tracts of axon conduction caused loss of the neurologic function. NSCs can differentiate into neurons, astrocytes, or oligodendrocytes, connect the spinal cord end, and rebuild neural pathways (Pereira et al., 2019; Vancamp et al., 2020; de Freria et al., 2021; Chen and Li, 2022). Several experimental studies have shown that acupuncture can induce the proliferation and differentiation of NSCs, thereby promoting the repair of injured nerves; however, the mechanism remains unclear (Tao et al., 2010; Zhang et al., 2013; Jiang et al., 2016; Dubrovsky et al., 2020).

Various hypotheses have been proposed to illustrate the acupuncture mechanism on NSCs. First, acupuncture could promote nerve regeneration and synaptogenesis by regulating the microenvironment of NSC transplantation and promoting SCI recovery (Tang et al., 2020c; Zhao et al., 2020; Yang et al., 2021). Second, electroacupuncture promotes the proliferation and differentiation of endogenous NSCs by regulating numerous endogenous signals. The upregulation of exosomal miR-146b, NeuroD1, the activation of the Notch pathway, and the downregulation of the PTEN expression are associated with acupuncture-induced improvement of neurological injury after ischemic stroke (Tao et al., 2014; Zhao et al., 2015; Sha et al., 2019; Zhang et al., 2020). In contrast, the potential signals of the acupuncture-induced NSC regulation in the SCI model include Wnt/β-catenin (Zhang et al., 2017a), ERK (Xu et al., 2019), miR-449a (Zhu et al., 2017), and Notch pathway (Wang et al., 2021b). Third, electroacupuncture reinforces the survival and synaptogenesis of transplanted NSC-derived neural network scaffolds as a neuronal relay bridging two severed ends of the injured spinal cord (Jin et al., 2019). Similarly, two other studies have shown that electroacupuncture facilitates the integration of the mesenchymal stem cell (MSC)–derived neural network into the transected spinal cord by elevating neurotrophin-3 (NT-3) (Ding et al., 2013; Yang et al., 2021). Moreover, pre-induction with NT-3 and retinoic acid after SCI before electroacupuncture could also promote the survival and differentiation of the grafted MSCs in gelatin sponge scaffolds (Zhang et al., 2014).

NT-3 is tightly associated with SCI recovery as the primary type of neurotrophic factor (Ding et al., 2009; Mo et al., 2016; Tu et al., 2017b). Electroacupuncture promotes the intrinsic growth ability of spinal neurons after SCI by activating the calcitonin gene-related peptide/α-calcium/calmodulin-dependent protein kinase/NT-3 pathway (Xu et al., 2021b). Additionally, electroacupuncture treatment can promote the differentiation and remyelination of MSCs and oligodendrocyte precursor cells, protect spinal motor neurons, and alleviate muscle atrophy after SCI, along with elevation of the NT-3 expression (Huang et al., 2011; Yan et al., 2011; Ding et al., 2015; Liu et al., 2015; Zhang et al., 2017b).

## Summary and Prospects

**FIGURE 1:**
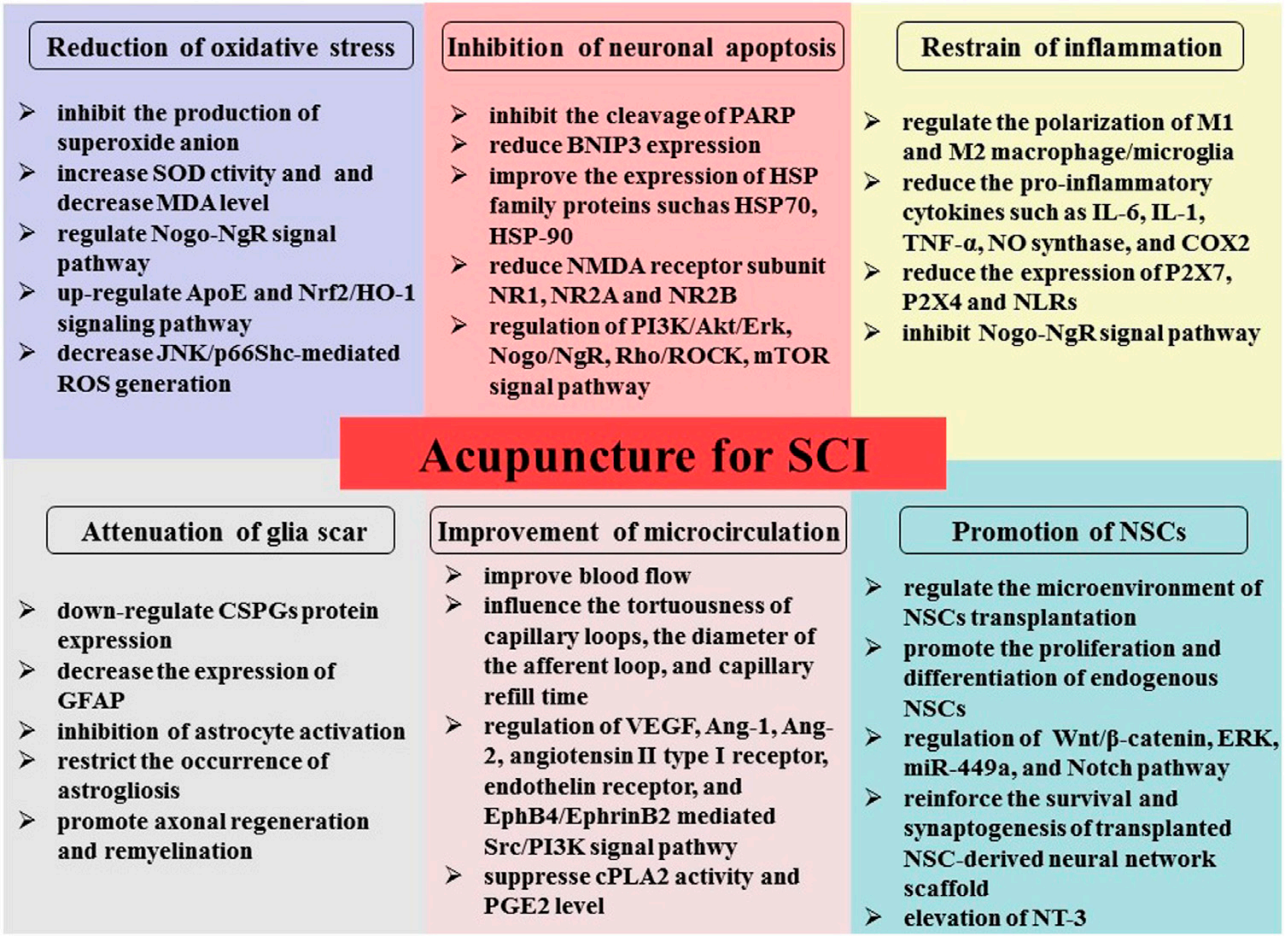
Illustration of the possible mechanism underlying acupuncture therapy in SCI, including oxidative stress reduction, inflammation and apoptosis inhibition, microcirculation improvement, reduction of glial scar formation, and stimulation of NSC differentiation and proliferation.

SCI is characterized by high mortality and disability, with complex regeneration and repair. We explained in detail the underlying mechanisms of acupuncture therapy for SCI, including oxidative stress reduction, inflammation and apoptosis inhibition, microcirculation improvement, glial scar formation reduction, and stimulation of NSC differentiation (Figure 1). This review could provide an experimental basis for better clinical application of acupuncture in SCI. However, SCI has complex pathophysiology. Therefore, significant research should be focused on the pathogenesis of acupuncture therapy to formulate mechanism-based specific intervention strategies and help SCI patients achieve better outcomes and recovery of impaired neurological function.

Although this review primarily summarizes recent preclinical studies, acupuncture clinical trials for SCI have shown positive results. Acupuncture alleviates the neurogenic bladder (Cheng et al., 1998; Honjo et al., 2000), chronic shoulder pain (Dyson-Hudson et al., 2001; Dyson-Hudson et al., 2007), neuropathic pain (Norrbrink and Lundeberg, 2011; Estores et al., 2017), and osteoporosis (Meng et al., 2014) and improves neurological (sensory and motor) functions (Wong et al., 2003). Interestingly, a study that enrolled seven healthy volunteers and three cervical SCI patients observed that the functional magnetic resonance imaging (fMRI) technique detected an activation centered at C6 and C2 cervical spinal cord levels by using acupuncture at L4 and L11, proving the existence of the meridians and points. An fMRI can be used as a harmless research and monitoring method to explore the effect of acupuncture therapy on SCI patients (Chen et al., 2007). However, most clinical trials are single-center trials with few subjects and are not conducted in a double-blinded manner.

Acupuncture can be an emerging therapy for the treatment of SCI as a simple, safe, and low-risk treatment. Although many basic studies and clinical trials have established the advantages of acupuncture in SCI, large-scale and multi-centric clinical trials are needed to authenticate the effect further. Moreover, the concept of precision medicine could further explore the best indicators in acupoint selection, stimulation frequency, starting time, and duration, for achieving individualized treatment. Thus, modern analytical techniques should be used to quantitatively analyze the variations in physiological and pathological indexes after acupuncture, which could popularize the global application of acupuncture.
