# Erratum: Mechanism Underlying Acupuncture Therapy in Spinal Cord Injury: A Narrative Overview of Preclinical Studies

**DOI:** 10.3389/fphar.2022.947461

**Published:** 2022-06-14

**Authors:** Frontiers Production Office

**Affiliations:** Frontiers Media SA, Lausanne, Switzerland

**Keywords:** acupuncture, spinal cord injury, therapy, mechanism, apoptosis, inflammation, oxidative stress, neuroprotection

Due to a production error, the corresponding author for this published article was erroneously listed as “Kungpeng Jiang”. The correct corresponding author is “Xinle Chen, 178451229@qq.com”. The publisher apologizes for this mistake.

